# COVID-19 and Cardiac Arrhythmias: A Review of the Literature

**DOI:** 10.7759/cureus.17797

**Published:** 2021-09-07

**Authors:** Abdul Rahman Akkawi, Mohamad Ghazal

**Affiliations:** 1 Internal Medicine, American University of Beirut, Beirut, LBN; 2 Internal Medicine, American University of Beirut Medical Center, Beirut, LBN

**Keywords:** cardiac arrhythmias, cardiovascular diseases, heart diseases, covid-19, covid-19-induced arrhythmia

## Abstract

Severe acute respiratory syndrome coronavirus 2 (SARS-CoV-2) is the cause of the ongoing coronavirus disease 2019 (COVID-19) pandemic. There are many documented COVID-19-related cardiac complications, one of the most feared is arrhythmia. Many ongoing studies are evaluating the pathophysiology of COVID-19-induced arrhythmia. However, our knowledge about the exact mechanism of the latter is still limited. The underlying possible mechanisms could be related to direct or indirect endomyocardial tissue damage. It is also noted in several studies that cardiac arrhythmias are the consequence of systemic illness, proarrhythmic medications, and electrolytes imbalances in hospitalized patients and not solely the direct effects of COVID-19 infection. In this review article, we present the different aspects of arrhythmias in COVID patients, possible associated conditions, and triggers.

## Introduction and background

Severe acute respiratory syndrome coronavirus 2 (SARS-CoV-2), which causes coronavirus disease 2019 (COVID-19), has recently emerged and grown into a global pandemic. SARS-CoV-2 belongs to the Coronaviridae subfamily which contains several other coronaviruses such as the Middle East Respiratory Syndrome coronavirus (MERS-CoV) [1]. Coronaviruses are named after the spikes on their surface which form a crownlike dome, and they are known to cause respiratory infections in humans and animals [2]. SARS-CoV-2, in particular, can lead to severe, and sometimes lethal, respiratory infections in humans [3]. This has created multiple challenges since acute respiratory infections are one of the known triggers for cardiovascular diseases (CVD), and the presence of CVD may complicate and worsen the course of the infectious disease [4,5]. SARS-CoV-2 binds to the zinc peptidase angiotensin-converting enzyme 2 (ACE2), which acts as a receptor for the virus [6]. ACE2 is a surface molecule found on vascular endothelial cells, arterial smooth muscle, and cardiac myocytes [7,8]. When SARS-COV2 attaches to ACE2 receptors on myocardial cells, it will cause their downregulation. This will result in angiotensin II accumulation and consequently adverse myocardial remodeling mediated by its action on ACE1 receptors [9]. Several cardiac complications, including new or worsening arrhythmias, are common in pneumonia patients due to COVID19 [6].

## Review

Studies have shown that the pathophysiology of arrhythmia in COVID-19 could be the result of tissue damage through myocarditis or myocardial infarction. Another reason for arrhythmia includes right ventricular strain secondary to pulmonary hypertension or pulmonary embolism. Moreover, arrhythmia can also be caused by cell-mediated cytotoxicity by CD8+ T lymphocytes that migrate into the heart and cause myocardial inflammation. This is mainly driven by the over-activation of lymphocytes due to cytokine storm resulting in the excessive release of proinflammatory mediators causing a positive feedback loop of immune activation and myocardial injury. Other possible mechanisms of arrhythmia are the use of proarrhythmic drugs in the management of COVID, electrolytes imbalances in hospitalized patients, and endogenous catecholamine adrenergic status [10]. Several recent studies have reported the presence of arrhythmias among patients with COVID-19. In a case series by Liu et al., palpitations were reported as the initial symptom in 10 (7.3%) out of 137 COVID-19 patients presenting to tertiary hospitals in the Hubei province in January 2020 [11].

A study by Guo et al. showed that patients with underlying CVD exhibited elevated levels of troponin-T (TnT), which led to more frequent development of complications including malignant arrhythmias and ventricular tachycardia/fibrillation [12]. Of interest, however, is the fact that patients without previous history of CVD also expressed high levels of TnT, and also developed malignant arrhythmias, albeit at a lower frequency (TnT levels were elevated in 13.2% of patients without underlying CVD vs. in 54.5% of patients with CVD; malignant arrhythmias occurred in 5.2% of patients without CVD vs. 11.5% in patients with CVD). Cardiac arrhythmias were two-fold more frequent at elevated Troponin levels.

In a global case series conducted in 29 institutions across the world, 827 out of 4526 hospitalized COVID-19 patients developed an arrhythmia [13]. The most common of which was Atrial fibrillation presenting in 80% of these patients; 20.7% developed ventricular arrhythmias, and 22.6% had bradyarrhythmia [13]. Furthermore, it was shown that arrhythmias were associated with high morbidity and mortality among those patients: 43% of patients who developed arrhythmia were mechanically ventilated and 51% survived hospital discharge [13].

In another study by Wang et al., 138 hospitalized patients with COVID-19 were observed. Of those patients, 23 (16.7%) developed arrhythmias, and 16 had to be transferred to the intensive care unit (ICU) due to this complication [14]. Cardiac arrhythmias were more common in patients requiring ICU admission (44.4% vs 6.9%). COVID-19 has been reported to cause myocardial injury according to the mechanisms discussed above. In most cases, myocardial damage appeared to be caused by increased cardiometabolic demand associated with the systemic infection, the electrolyte abnormalities, and the ongoing hypoxia caused by severe pneumonia or ARDS. These factors can potentiate cardiac arrhythmias. It is quite possible that the virus may lead to atrial or ventricular arrhythmias following cases of fulminant myocarditis with cardiogenic shock [15,16].

Bhatla et al. evaluated the clinical records of a cohort of 700 patients hospitalized with COVID-19 between March 6, 2020, and May 19, 2020. Throughout hospitalization, a total of 53 arrhythmic events occurred. Of those, 9 patients experienced cardiac arrest (6 cases of pulseless electrical activity, 1 episode of torsades de pointes, and 2 asystole events), and 25 experienced incident atrial fibrillation events that required management with diltiazem and amiodarone. In addition, there were 10 events of non-sustained ventricular tachycardia, and 9 clinically significant bradyarrhythmia [17]. However, the findings of this study seem to suggest that the incidence of arrhythmic events in patients with COVID-19 is not solely the consequence of the viral infection, but rather due to a combination of factors, namely the severity of the disease course. In Italy, there was a 58% increase in out-of-hospital cardiac arrest during the 40 days of the COVID-19 outbreak in comparison with the same period in 2019.

A survey organized by the Heart Rhythm Society (HRS) and sent to more than 1100 cardiac electrophysiology specialists around the world showed that atrial fibrillation was the most common arrhythmia in hospitalized patients with COVID presenting in 142 (21%), atrial flutter by 37 (5.4%), sustained atrial tachycardia by 24 (3.5%), and paroxysmal supraventricular tachycardia by 39 (5.7%) out of 683 respondents [18]. Ventricular arrhythmias were also reported, the most common form was monomorphic premature ventricular contractions reported by 36 (5.3%). Other ventricular arrhythmias were also reported: polymorphic premature ventricular contractions, non-sustained ventricular tachycardia (VT), sustained monomorphic VT, polymorphic VT/Torsade de Pointes, VT/ventricular fibrillation (VF) arrest, and pulseless electrical activity [18]. Regarding bradycardias in COVID-19 patients, of 663 respondents, sinus bradycardia and complete heart block were the most reported by 51 (8%) and 51 (8%) respectively. First- or second-degree AV block, bundle branch block, or intraventricular conduction delay were also reported in those patients [18].

Kochav et al. detailed a series of cases in which various cardiac arrhythmias were associated with COVID-19 infection and treatment [19]. The occurrence of these arrhythmias did not correlate with the severity of lung injury on a chest X-ray. Among these arrhythmias were high-grade atrioventricular block, atrial fibrillation, polymorphic ventricular tachycardia, and cardiogenic shock with pulseless electrical activity. The fourth case was likely due to fulminant myocarditis complicated by sudden AV block without an escape rhythm, demonstrating how rapid hemodynamic decline can be in the setting of COVID-19 infection [19]. Table 1 lists the potential direct and indirect mechanisms of cardiac arrhythmias in the setting of COVID-19.

**Table 1:** Mechanisms of cardiac arrhythmias in the setting of COVID-19.

Mechanisms of cardiac arrhythmias in the setting of COVID-19
Direct viral damage to the myocardial cells and/or conduction system
Worsening of pre-existing myocardial function or conduction disturbances
Right ventricular strain due to pulmonary hypertension or pulmonary embolism
Electrolyte abnormalities
Drug effect for COVID-19 management and interactions with other medications
Acute coronary syndrome with ongoing ischemia
Adrenergic stress leading to electrical instability including atrial fibrillation
Inflammation and electrophysiological effects of cytokines (particularly IL-1, IL-6, TNF-α) modulating the expression and/or function of several potassium and calcium channels as well Connexin 43 and increasing the QT interval/Torsades de pointes susceptibility
Hypoxia-induced and acidosis-induced

SARS-CoV-2 is a relatively recent virus that has taken the world by storm. With the basic reproduction number (R0) of the virus being 2-3, which means that every infected person has the potential to transmit the virus to two to three other individuals, the exponential growth of cases has been observed [20]. Wang M. et al. study has shown that the antiviral drug Remdesivir effectively inhibits SARS-CoV-2 in-vitro [21]. However, Wang et al. showed that although patients receiving remdesivir had a faster clinical improvement than those receiving placebo, the difference was not statistically significant [22]. Moreover, studies have shown a lack of benefit when treating COVID-19 patients with chloroquine, hydroxychloroquine, or azithromycin; therefore, they are not recommended as standard therapy for those hospitalized with COVID-19 as they may increase the risk of arrhythmias.

## Conclusions

To conclude, COVID-19 is associated with an increased risk of arrhythmia in hospitalized patients, the most common of which is atrial fibrillation, and this could be the result of direct or indirect myocardial tissue damage through multiple mechanisms including the use of proarrhythmic drugs (chloroquine, hydroxychloroquine, or azithromycin). These drugs have shown a lack of benefit in COVID-19 treatment therefore their use should be prohibited. Future investigations regarding the mechanism of arrhythmias in COVID-19 patients may aid in our understanding of the pathophysiology of arrhythmia and will help us prevent such complications and guide our treatment in this population.
